# Obesity‐induced immunological effects on the skin

**DOI:** 10.1002/ski2.160

**Published:** 2023-02-28

**Authors:** Janani A. Palanivel, George W. M. Millington

**Affiliations:** ^1^ Derme Cure Skin and Cosmetic Clinic Chennai India; ^2^ Dermatology Department Norfolk and Norwich University Hospital Norwich UK

## Abstract

There is an increasing prevalence of obesity globally. Equally, the significance of maintaining a healthy body weight for maintaining a healthy skin homoeostasis is gaining greater attention. On this background, there is growing evidence of an adverse influence of excess body weight on the immune system, which has a resultant detrimental effect on the functioning of the skin. The presence of obesity appears to intensify various inflammatory skin disorders. These immune‐dermatological consequences in the obese occur because of multiple adverse changes in the skin physiology, endocrine imbalance, metabolic deviations, alterations in circulation, skin microbiome and immunological disruptions. The purpose of this article is to highlight the profound impact of increased fat deposition on cutaneous immunology and its role in the pathophysiology of various chronic inflammatory dermatological conditions. Understanding these immunological modulations will aid in developing therapies targeting the specific inflammatory mediators in the management of obesity‐associated chronic immunological skin disease.

## Graphical abstract

Several inflammatory dermatoses are aggravated by obesity. The skin manifestations in obese individuals occur due to various changes in skin physiology, endocrine imbalance, metabolic deviations, changes in circulation, skin microbiome and immunological disruptions. The purpose of this article is to highlight the profound impact of increased fat deposition on cutaneous immunology and its role in the pathophysiology of various chronic inflammatory dermatological conditions.

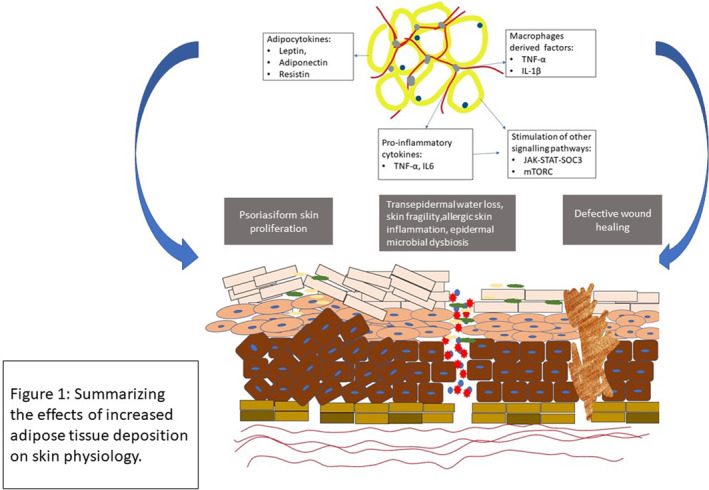

1

**What is already known about this topic?**

- Obesity is known to be associated with a number of inflammatory dermatoses.

**What does this study add?**

- The purpose of this article is to highlight the profound impact of increased fat deposition on cutaneous immunology and its role in the potential pathophysiology of various chronic inflammatory dermatological conditions.

## 2 INTRODUCTION

In a data analyzing the obesity trends world‐wide between 1980 and 2015, high body mass index (BMI) contributed directly to 4.0 million deaths globally each year.^1^ Of these deaths, nearly 70% were due to cardiovascular disease and 60% of these occurred in obese individuals.^1^

**FIGURE 1:**
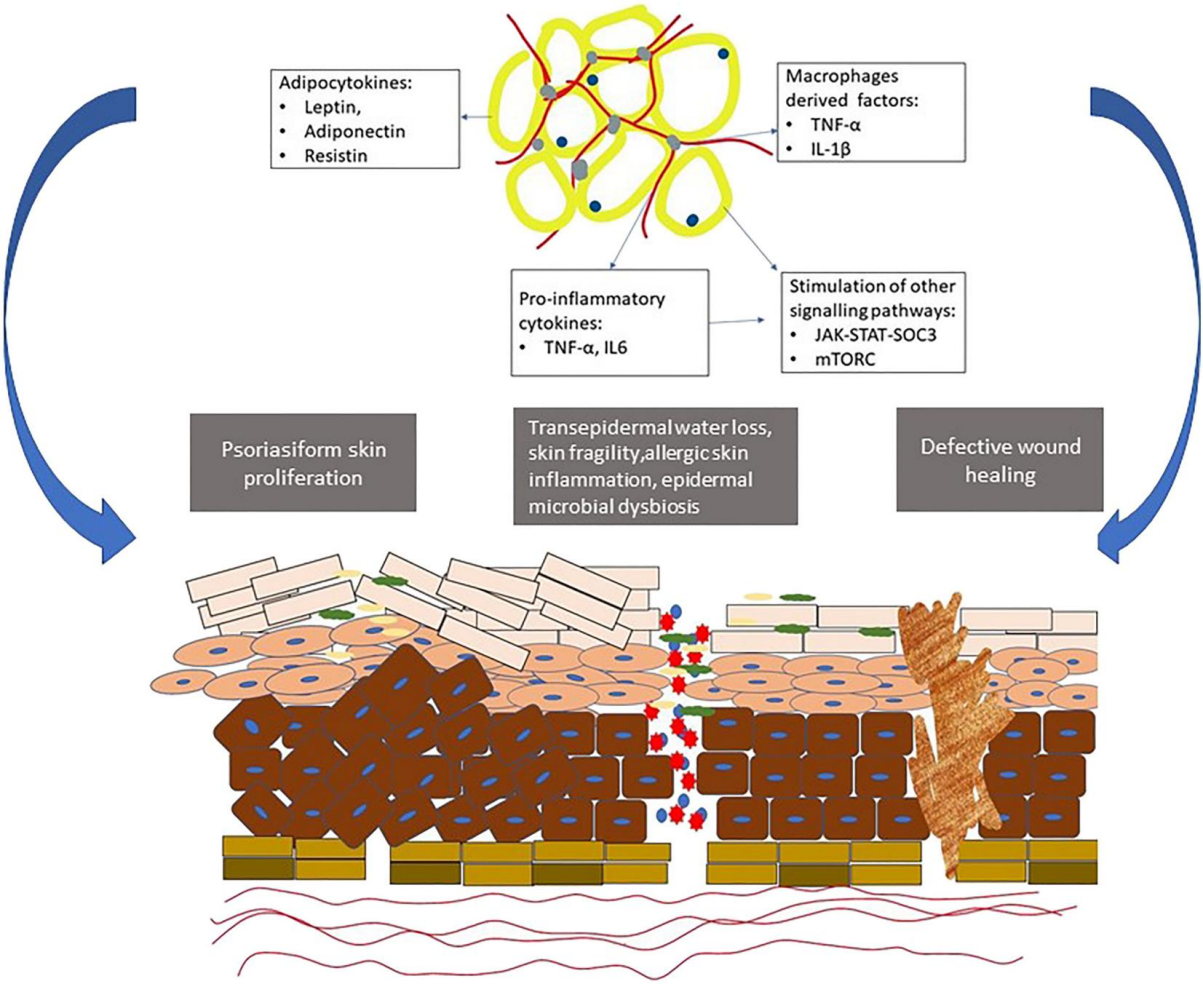
Summarizing the effects of increased adipose tissue deposition on skin physiology

Besides secondary metabolic disorders (such as insulin resistance, leading to type‐2 diabetes mellitus), obesity also directly interferes with natural skin functioning through hormone dysregulation, lymphatic disturbance and thickening of the skin folds.^2^ There is growing evidence for an association between obesity and inflammatory dermatoses such as psoriasis, hidradenitis suppurativa and atopic dermatitis (AD) (Table 1, Figure 1).^2^, ^3^

**TABLE 1:** Skin complications of obesity where immune dysfunction may be relevant

Alterations in the cutaneous microbiome
Poor wound healing
Cutaneous infection
Lymphovascular disorders and ulceration
Psoriasis
Intertrigo
Atopic eczema
Hidradenitis suppurativa
Pilonidal sinus
Irritant contact dermatitis
Scleroedema
Livedo reticularis

In this review, we discuss the potential mechanistic link between fat deposition and skin immunology (Table 2). We also discuss other recently explored immunological links between obesity and inflammatory skin diseases, such as the skin microbiome. There may be some genetic factors in these associations. For example, proopiomelanocortin (POMC) deficiency is a monogenic obesity syndrome, associated with tall stature, fair skin and adrenal insufficiency, with severe and potentially fatal immune disruption.^19^, ^20^, ^21^ Other monogenic syndromes involving obesity, immune‐alteration and the skin may also exist, but discussion of these is beyond the remit of this article.^19^

**TABLE 2:** Obesity‐induced skin immunological and structural changes

Skin structural changes due to obesity	Obesity‐induced immune alteration
Psoriasiform skin inflammation	Increased expression of leptin stimulation‐induced pro‐inflammatory cytokines IL‐17A and IL‐22 on human keratinocytes.^4^, ^5^, ^6^
	Increased leptin, resistin, TNF‐α, IL‐6, and decreased adiponectin levels.^6^, ^7^
	Aberrant mTOR activity causing increased keratinocyte proliferation.^8^
	Activated JAK‐STAT‐SOC3 signalling.^9^
Atopic skin diseases	Increased IL‐6, TNF‐α, leptin and decreased adiponectin, resulting in the downregulation of Tregs.^10^
	Activated NF‐kB signalling pathway^10^
	Increased mTOR activity and decreased filaggrin expression.^11^
	Increased expression of SOC3 in atopic dermatitis.^12^
Defective wound repair due to defective collagen turnover.	Reduction in growth factors‐TGF‐β, PDGF, and IGF‐1.^13^, ^14^
	Increase in pro‐inflammatory cytokines such as TNF‐α and IL‐1β.^13^, ^14^
	Decreased tissue inhibitors of metalloproteinase activity.^13^, ^14^
Systemic lupus erythematosus	Increased circulating leptin and decreased adiponectin.^15^, ^16^
	Aberrant expression of IFN‐γ.^17^
Increased lipid synthesis, cell proliferation and inflammation in hidradenitis suppurativa	Increased mTOR1 and PI3K‐Akt signalling.^18^

Abbreviations: IL, interleukin; JAK‐STAT, Janus Kinase‐signal transducers and activators of transcription; mTOR, mammalian target of rapamycin; NF‐kB, nuclear factor‐kappa B; Tregs, Regulatory T‐lymphocyte; PGDF, platelet derived growth factor; PI3K‐Akt, phosphatidylinositol 3‐kinases, protein‐kinase B; PPARδ, peroxisome proliferator‐activated receptor; SOC3, suppressor of cytokine signalling 3; TNF, tumour necrosis factor.

## 3 ADIPOSE TISSUE BIOLOGY

Morphologically adipose tissue exists as white adipose tissue (WAT), brown adipose tissue (BAT) and beige adipose tissue in different depots.^22^ In adults, WAT is present in distinct depots ‐ underneath the skin (subcutaneous), intra‐abdominal (visceral) and in reticular dermis (dermal WAT). The WAT is responsible for insulation, energy storage, and secretion of regulatory peptides called adipokines.^22^ Adipokine release varies according to subcutaneous fat tissue type, its distribution and energy status, with diverse paracrine and endocrine effects on metabolic and immune functions.^22^ Adipose tissue disorders are therefore associated with cardiac, metabolic, inflammatory conditions and cancer.^22^, ^23^

Brown adipose tissue also produces adipokines, but its principal roles include fatty acid metabolism and heat production.^22^, ^23^ Brown adipose tissue is predominantly found in neonates, but adults also have minor discrete BAT deposits in their upper body subcutaneous fat, which could be metabolically relevant.^22^, ^23^ Beige adipose tissues have functional similarities to BAT, but they are dispersed amongst WAT.^22^ Beige adipocytes originate from WAT progenitors.^22^, ^23^ An increased ratio of beige adipose tissue to WAT may be beneficial metabolically.^22^, ^23^

The dermal WAT (dWAT) is a thin layer of fat distinct from the subcutaneous fat under the dermis.^22^ In the mice and rodents, dWAT is demarcated from the subcutaneous tissue by a sheet of thin layer of skeletal muscle‐*panniculus carnosus*.^23^, ^24^ However, in humans, this muscle is rudimentary, therefore in humans, dWAT exists in contact with the subcutaneous fat tissue deposited in the reticular dermis.^23^, ^24^

The dWAT develops independently and has its own separate non‐metabolic function.^24^ The dWAT may influence the hair cycle and wound regeneration.^23^ The production of dWAT is linked with signals from growing hair follicles, cold stress and bacterial infections, suggesting an innate immune defence function.^24^

This concise review discusses the immunological interference of aberrant adipose deposition on skin health.

## 4 ROLE OF ADIPOKINES DYSREGULATION AND SKIN INFLAMMATION

Uncontrolled adipocyte hypertrophy results in an imbalance between secretion of pro‐inflammatory cytokines and anti‐inflammatory cytokines, thus resulting in an immune disruption.^4^

Adipokines such as leptin, adiponectin, interleukin (IL) 6, tumour necrosis factor‐alpha (TNF‐α) and plasminogen activator inhibitor (PAI‐1) from adipose tissue, are the main determinants of obesity‐induced inflammation.^4^, ^5^ Serum leptin levels are elevated in obese individuals and it may be one of the factors linking obesity and psoriasis.^4^ For example, increased leptin signalling stimulates the release of pro‐inflammatory mediators‐interleukin (IL‐1, IL‐6, IL‐17) and tumour necrosis factor (TNF‐α),^5^, ^6^ which can promote keratinocyte proliferation, epidermal and dermal hyperplasia and angiogenesis.^25^

Numerous studies have shown leptin deficiency as a contributory link between psoriasis and obesity.^26^, ^27^, ^28^, ^29^ In an in vivo study, leptin deficiency attenuated imiquimod‐induced psoriasis‐like skin inflammation in a leptin‐deficient (*ob/ob*) mouse model.^26^ Similarly, in vitro, the leptin stimulation‐induced pro‐inflammatory proteins such as IL‐17A and IL‐22 in human keratinocytes.^26^ These findings were replicated in human studies, where serum leptin, tissue leptin and leptin receptor expression were higher in psoriasis patients than healthy controls.^27^ Looking at the expression of leptin messenger (mRNA) in subcutaneous adipose tissue (SAT) in a group of obese and non‐obese psoriasis patients, the PASI score, serum leptin and expression of leptin mRNA in SAT was higher in the obese group compared to the non‐obese group.^28^ A similar association is established in psoriatic arthritis patients, where leptin levels are also increased.^29^

Interestingly, leptin association in promoting hair growth was established in a mouse model.^30^ In this study with a leptin‐deficient mouse (the *ob/ob* mouse model), the period of the first anagen cycle was significantly delayed.^30^

Some data suggest that elevated levels of other adipocytokines namely visfatin and resistin, may be linked with more severe psoriasis.^29^ In particular, increased visfatin levels may be associated with increased cardiovascular morbidity in psoriasis.^29^

Similarly, lower levels of adiponectin (an anti‐inflammatory cytokine) may correlate with the severity of psoriasis.^7^ The blood levels of adiponectin are decreased in patients with both psoriasis^7^, ^31^ and obesity.^32^ In an in vivo study reduced adiponectin levels in mice, showed increased psoriasiform skin lesions with enhanced infiltration of IL‐17 in the dermal cells,^33^ In a case‐control study including 50 patients with psoriasis and 50 healthy individuals, the serum levels of adiponectin was evaluated with ELISA.^34^ Serum levels of adiponectin were significantly higher in control than in psoriasis patients.^34^ However, there was no correlation between adiponectin levels and the psoriasis severity index score (PASI).^34^

In another in vivo study with obese‐diabetic mice, as well as psoriasiform skin lesions, liraglutide (an anti‐diabetic and weight loss medication) reduced the expression of inflammatory cytokines—IL‐23, IL‐17A, IL‐22, and TNF‐α.^35^ Thus, this study suggests there may be a relationship between obesity, diabetes and psoriasis.^35^ Similarly, IL‐6 is elevated in those with obesity, as well as in patients with psoriasis.^36^, ^37^ IL‐6 is essential for differentiation of T‐helper cells (Th‐17) cells and pathogenesis of psoriasis, through facilitation of keratinocyte growth, activation and neutrophil differentiation.^38^

In addition to the adipocytokines, stromal vascular cells (comprising mesenchymal cells, vascular endothelial cells, nerve cells, macrophages, T cells, and B cells) from visceral and subcutaneous tissue, have a role in mediating chronic inflammation.^39^, ^40^ Macrophages activated in fat tissue can trigger psoriasis‐inducing inflammatory cytokines such as IL‐17 and TNF‐α.^41^ The adipose tissue macrophages in obese and type 2 diabetic individuals release IL‐1β, which in turn promotes the production of T cell cytokines—IL‐17 and IL‐22.^42^ This generates pathogenic Th17 cells that promote autoimmunity.^42^, ^43^

An analogous association was noted with obesity and atopic eczema. A BMI age percentile greater than or equal to 95 correlates with a higher incidence of AD.^44^ A case control study from the United States, with 86 969 children with AD and 116 564 matched controls, found that AD was significantly associated with metabolic syndrome and obesity.^45^ Nevertheless, in another longitudinal study involving 10 611 children, 1834 children had AD associated with lower height and higher BMI.^46^ However, the association between AD and height decreased by age 14. Similarly, the association between AD and BMI diminished by 5.5 years 46 Thus, the authors concluded that the initial short stature could have been the link, rather than the BMI.^46^

Disturbed adipocytokines levels also correlate with atopic eczema.^10^ This may be due to the interleukin‐6 (IL‐6), TNF‐α, leptin, and decreased adiponectin, resulting in the downregulation of regulatory T‐lymphocytes (Tregs).^10^ In addition, activate the nuclear factor‐kappa B (NF‐kB) signalling pathway, inducing inflammation.^10^ The elevated pro‐inflammatory cytokines can reach the intestine through the bloodstream and affect the intestinal barrier and microbiome.^10^ This may predispose obese individuals to food allergies.^10^ Though evidence linking obesity and atopic eczema is inconsistent, the role of adipose deposition and inflammatory influence on the skin cannot be ignored.

Similarly, an increased circulating leptin and decreased adiponectin can influence systemic lupus erythematosus (SLE) severity.^15^, ^16^, ^29^ Enhanced leptin levels could be a factor triggering autoreactive T‐lymphocytes and expansion of Th17 cells in lupus‐prone mice.^29^ In addition, leptin may activate the phosphatidylinositol 3‐kinase/protein kinase B (PI3K/AKT) signalling pathway in SLE patients.^47^ Similarly, aberrant expression of interferon (IFN) ℽ alters the host immune response and influences the development of systemic autoimmune diseases, such as SLE and rheumatoid arthritis.^17^

Obesity and type 2 DM are connected with poor wound healing due to the accumulation of inflammatory cells in the wound site, as well as other mechanisms, such as increased risk of cutaneous infections and peripheral neuropathy.^13^ Wounds heal less well in the obese.^48^ Factors could include vascular insufficiency, oxidative stress and nutritional deficiencies (poor diet).^48^ In addition, delayed wound healing with obesity may be due to dysregulation of immune mediators.^48^

Adipose tissue macrophage infiltration in obesity contributes to insulin resistance and pathological inflammatory changes.^49^ Adipose tissue macrophages shift from an anti‐inflammatory M2 polarized state to a proinflammatory M1 state in diet‐induced obese mice.^49^ M2 state macrophages are vital in tissue repair and homoeostasis, which is affected in obesity.^49^ In addition, delayed wound healing occurs due to a reduction in several growth factors, including TGF‐β, platelet‐derived growth factor (PDGF), and insulin‐like growth factor (IGF)‐1 and increased pro‐inflammatory cytokines such as TNF‐α and IL‐1β.^13^, ^14^ Wound healing is affected due to dysregulated collagen turnover and impaired collagen deposition caused by decreased tissue inhibitors of metalloproteinase activity.^13^, ^14^

Adiponectin stimulates angiogenesis, and this is decreased in obesity, which affects wound healing.^32^, ^50^ For example, in adiponectin knock‐out mice, the angiogenic repair of the ischaemic hind limb is impaired.^51^ Similarly, wound closure is delayed in adiponectin‐deficient mice compared to wild‐type mice, as the keratinocyte migration and proliferation was impaired. Administration of adiponectin (systemic and topical) accelerated the wound healing in adiponectin deficient and diabetic db/db mice.^50^

In a retrospective analysis in diverticulitis surgery patients, obese patients had higher frequencies of sepsis, than in those with a more normal BMI.^52^ Likewise, in a large prospective observational study in critically ill surgical patients documented significantly more catheter‐related infections and other bloodstream infections in obese versus non‐obese patients.^53^ In another study comparing obese patients and non‐obese individuals who underwent elective breast surgery, the obese had a 12 fold increased risk of post‐operative complications, compared with the non‐obese.^54^

Thus, there is a large array of evidence linking obesity and wound and other infections. In addition to the expected surgical complications, an immune‐mediated response in obese individuals will affect the wound repair.

## 5 JAK‐STAT SIGNALLING AND SKIN INFLAMMATION

Janus Kinase (JAK)‐signal transducers and activators of transcription (STAT) play a vital role in immune development, homoeostasis, and metabolism.^9^, ^12^, ^55^ Leptin resistance occurs in obesity, due to chronic activation of JAK‐STAT signalling.^12^, ^55^ In the CNS, increased leptin function and peripherally increased IL‐6 function activates JAK‐STAT signalling.^9^ Chronic activation of JAK‐STAT in obesity stimulates the suppressor of cytokine signalling 3 (SOC3). SOC3 is a negative feedback regulator of leptin.^12^

Thus, chronic inhibition of leptin results in leptin resistance and inability to induce satiety and increase energy expenditure.^34^ Chronically elevated IL‐6 due to increased secretion from adipose tissue leads to increased SOC3 levels in primary insulin‐sensitive peripheral tissues‐white adipose tissue, liver, and muscles.^12^ It mediates decreased insulin sensitivity and results in insulin resistance, fatty liver and lipogenesis.^12^

The role of JAK‐STAT‐ SOC3 signalling is well‐established in immune‐mediated diseases such as AD.^9^ Similarly, IL‐6, when synthesized transiently, plays a vital role in host immune response to infections or injuries.^56^, ^57^ Once the stress is removed, IL‐6 mediated action is terminated by negative regulatory systems.^56^, ^57^ However, a dysregulated and persistent IL‐6 production is linked with autoimmune skin diseases such as psoriasis and systemic lupus erythematosus.^56^, ^57^

Thus, there exist a correlation between JAK‐STAT‐SOC3 and IL‐6 signalling in obesity and chronic inflammatory skin diseases which is less explored. Understanding this link will serve as a future therapeutic target for persistent inflammatory skin disorders.

## 6 m‐TORC SIGNALLING AND SKIN INFLAMMATION

The mammalian target of rapamycin (mTOR) signalling complex, is a nutrient and energy sensor, vital for protein synthesis, cell differentiation, growth and proliferation.^58^ It exists as two distinct complexes—mTOR1 and mTOR2.^58^ The two complexes bind to different substrates and differ in activity to enhance protein synthesis within the cell.^57^ mTOR1 is regulated by insulin, growth factors, and certain amino acids.^58^ The mTOR1 consists of multiple components such as the regulatory associated protein of mTOR (Raptor), upstream modulators such as phosphatidylinositol 3‐kinases (PI3Ks), protein kinase B (Akt).^58^

Overloading of carbohydrates, fat, and proteins leads to obesity characterized by adipogenesis, perhaps due to activation of mTOR1.^59^ Insulin is a regulator of the mTOR complex and insulin resistance in obese rats stimulated the mTOR1 complex in the liver and muscle tissue, through increased expression of PI3Ks/Akt signalling.^59^

Alterations in the mTOR1 pathway may be associated with inflammatory skin diseases.^11^, ^18^, ^59^, ^60^ Under normal conditions, mTOR1 signalling is deactivated when keratinocytes proliferate and differentiate. However, aberrant mTOR1 activity leads to enhanced proliferation and reduced differentiation as seen in psoriasis.^8^

Increased mTOR1 signalling activity is associated with decreased filaggrin expression in a cell‐culture model of AD.^11^ In keratinocyte cell cultures, the upregulation of mTOR1 or AKT1 short hairpin RNA knockdown reduced protease cathepsin H (CTSH) expression.^11^ Translated to an in vivo or clinical situation, these data suggest disrupted skin barrier function, consequent to reduced filaggrin processing.^11^

In a cell‐culture experiment, looking at insulin signalling in sebocytes, this resulted in activation of the PI3K/Akt and mTOR pathways.^18^ This would induce high protein and lipid synthesis, increased cell growth and proliferation, and inflammation.^18^ In a study using skin from hidradenitis suppurativa (HS) patients, there was increased expression of mTORC1 both on lesional and non‐lesional skin of HS patients.^60^ Similarly, HS lesions show overlapping features with psoriatic skin inflammation, which could be because of the involvement of mTOR cascade in the pathogenesis of both.^61^, ^62^

Increased PI3K/AKT/mTOR signalling and resistance to apoptosis is found in basal cell carcinomas and cutaneous squamous cell carcinomas.^63^, ^64^ Activation of PI3K signalling can occur in keratinocytes stimulated by ultraviolet (UV) radiation and aberrant inflammatory cytokines.^65^ The links of obesity with melanoma non‐melanoma skin cancer are tenuous at best.^65^

An increase in mTORC1 signalling is seen in the epidermis of both rosacea patients and mouse models with rosacea‐like inflammation.^66^ This study showed that mTORC1 deletion in the epithelium, and inhibition by its specific‐inhibitors, could prevent rosacea‐like inflammation in LL37‐induced rosacea‐like mouse model.^66^ The cathelicidin LL37 binds to toll‐like receptor 2 and stimulates the mTORC1 signalling and through positive feedback loop increases the expression of cathelicidin in keratinocytes.^66^ The mTOR signalling and skin inflammation is a complex network and will need further investigation the role of mTOR signalling inhibitors in hyperproliferative skin disorders.

## 7 OBESITY, THE SKIN AND GUT MICROBIOMES

The skin is a rich ecosystem that supports a diverse milieu of microorganisms in its folds, invaginations, and specialized appendages.^67^, ^68^ The nature of the cutaneous microbiome is influenced by a number of factors.^67^, ^68^, ^69^ These include gender, age, ethnicity, host and bacterial genomes, diet, hormonal and metabolic variation, and environmental factors such as climate change and accelerated urbanization.^67^, ^68^, ^69^

The moist, sebaceous, and dry areas of the skin form a habitat for different commensals.^67^ For example, the humid areas such as the toe and phalangeal webs favour *Corynebacterium* and *Staphylococcal* colonization, respectively.^67^ Sebum secretion on face, chest and back promotes the growth of *Cutibacterium* (formerly known as *Propionobacterium*), *Staphylococcus* and *Malassezia* yeast.^23^ Dry areas such as arms and legs are composed of *Cutibacterium*, *Staphylococcus*, *Gammaproteobacteria* and *Betaproteobacteria*.^67^

A diverse, healthy microbiota is a critical component in helping the skin host cells fight against other pathogenic organisms by secreting antimicrobial molecules and competing with nutritional resources.^67^, ^68^ The skin microbiota also interacts with the complex adaptive and innate immune system through constant signalling between host keratinocytes and the cutaneous immune networks.^67^, ^68^

Our understanding of the microbiome's role in skin inflammation has recently undergone a significant change.^69^ Both the skin microbiome and the gut microbiome are vital in regulating the immune response and maintaining homoeostasis.^67^, ^68^, ^69^ An alteration in the microbiome diversity or hyperproliferation of any microbial community (including both the skin and gut microbiome) contributes to disrupted epithelial integrity and immune dysregulation, resulting in cutaneous inflammation.^69^ For example, studies show that acne vulgaris patients have a distinct gut microbiome compared to healthy controls. They have a lower abundance of *Firmicutes* and increased levels of *Bacteriodes*. Similarly, *Clostridium*, *Clostridiales*, *Lachnospiraceae*, *and Ruminococcaceae* are lower in acne patients.^70^

Similarly, evidence suggests that 90% of AD patients show a hyperproliferation of *Staphylococcus aureus* in lesional and non‐lesional skin.^71^ But levels of *Cutibacterium*, *Corynebacterium*, *Streptococcus*, *Acinetobacter*, *Prevotella*, *and Malassezia are decreased*.^72^ The peptidoglycan of *S*. *aureus* can promote human cathelicidin LL‐37 and vascular endothelial growth factor (VEGF) expression in keratinocytes, which cause inflammation.^73^ In addition, there is an altered gut microbiome in children suffering from AD.^74^ The gut of atopic infants show higher levels of *Clostridium* and *Escherichia* compared to healthy controls.^75^, ^76^, ^77^ And lower levels of *Akkermansia*, *Bacteroidetes*, *and Bifidobacterium* were found in AD patients, compared to healthy controls.^78^, ^79^

In psoriasis, the lesional skin has an overabundance of *Proteobacteria*, *Streptococcus* and *Cutibacterium*.^80^ Psoriasis is associated with irritable bowel syndrome, ulcerative colitis and coeliac disease.^81^ Though the exact mechanism is still not understood, it is suspected that the pro‐inflammatory cytokines affects the intestinal wall integrity.^81^

The western diets comprising high‐glycaemic and high‐fat foods can alter the gut and skin microbiome composition, mediating skin inflammation.^82^ Consumption of dairy products, high sugary diets, chocolates, and saturated fats modifies the gut microbial community, thus triggering metabolic signals and acne.^82^ In a study of the skin microbiome of mice fed on a high‐fat diet, the authors showed that *Corynebacterium* was dominant on the skin of high‐fat‐fed mice, compared to those on a calorie‐neutral diet.^83^ Interestingly, it was recognized that *Corynebacterium* could trigger skin inflammation in obesity by facilitating the secretion of mycolic acid.^84^ A similar association was noted between BMI levels and skin microbiome in a study involving 822 human skin samples.^85^ The skin microbial diversity was affected by BMI.^85^ The microbial communities were different in underweight, overweight, and obese individuals.^85^ The *Corynebacterium* overabundance statistically correlated with increasing levels of obesity, as measured by BMI.^85^

In addition, the local commensal ecology dysregulation increases the risk of infections and defective wound repair.^86^ Rood et al, studied the microbiome of post‐caesarean surgical sites in obese and non‐obese women.^86^ The incision site in obese women showed significantly higher bacterial biomass.^86^ Phylotypes *Firmicutes*, *Bacteroidales* and *Clostridiales* predominated more than the commensals, such as *Actinobacteria*, *Staphylococcus* and *Cutibacterium*.^86^

Thus, obesity alters skin immunology indirectly through epidermal and gut microbial dysbiosis.^86^ Currently, pre‐and probiotics aim to improve the gut microbiome, which could indirectly help with skin inflammation.^87^ Further research may lead to promising therapeutics for chronic inflammatory skin diseases.

## 8 CONCLUSION

With obesity becoming a global epidemic, there is an increasing prevalence of inflammatory diseases. The link between obesity and chronic diseases such as cardiovascular diseases, diabetes mellitus, and liver diseases is well‐studied. With the growing prevalence of skin diseases in obese individuals, various studies are evolving, linking obesity and skin diseases. Obesity can influence multiple aspects of skin health (Figure 1), such as variation in the skin microbiome, epidermal barrier disruption, lymphatic and vascular dysfunction, and dysfunctional wound healing (Table 2). Besides, increased pro‐inflammatory cytokines due to fatty tissue accumulation are crucial in altering skin immunology.

Though the molecular mechanisms linking obesity and skin inflammation remain elusive, understanding this complexity will enhance future therapies targeting specific inflammatory mediators that induce chronic skin inflammatory disorders.

## AUTHOR CONTRIBUTIONS

**Janani A. Palanivel**: Conceptualization (Equal); Data curation (Equal); Formal analysis (Equal); Investigation (Equal); Methodology (Equal); Writing – original draft (Equal); Writing – review & editing (Equal). **George W. M. Millington**: Conceptualization (Equal); Data curation (Equal); Formal analysis (Equal); Investigation (Equal); Methodology (Equal); Writing – original draft (Equal); Writing – review & editing (Equal).

## CONFLICT OF INTEREST

George W. M. Millington is the Editor in Chief of Skin Health and Disease.

## ETHICS STATEMENT

Not applicable.

## Data Availability

The data that support the findings of this study are available from the corresponding author upon reasonable request.
